# Highlighting the Place of Metastasis-Directed Therapy in Isolated Liver Metastases in Prostate Cancer: A Case Report

**DOI:** 10.3389/fonc.2021.764758

**Published:** 2021-11-17

**Authors:** Anne-Emmanuella Yeo, Aurore Hendrix, Caterina Confente, Nicolas Christian, Baudouin Mansvelt, Géraldine Pairet, Emmanuel Seront

**Affiliations:** ^1^ Department of Medical Oncology, Jolimont Hospital, La Louvière, Belgium; ^2^ Department of Radiotherapy, Jolimont Hospital, La Louvière, Belgium; ^3^ Department of Surgery, Jolimont Hospital, La Louvière, Belgium; ^4^ Department of Pathology, Jolimont Hospital, La Louvière, Belgium

**Keywords:** metastasis-directed therapy, liver metastasis, oligometastatic, prostate cancer, abiraterone acetate, case report

## Abstract

Metastatic prostate cancer remains a challenge for clinicians. Metastases involve mainly the bone compartment and can manifest as oligometastatic disease. In this setting, the role of metastasis-directed therapies (MDT) including surgery and/or stereotactic body radiotherapy is currently evaluated. Visceral metastases are less common and have very poor prognosis in mPC. Whether treating isolated visceral metastases such as liver metastases with MDT could increase the prognosis remains unknown. We report the management of a prostate cancer patient who progressed on androgen deprivation therapy with apparition of two liver metastases. We describe the feasibility of combining MDT with abiraterone acetate and prednisone in a patient with metastatic castration-resistant prostate cancer. MDT allowed the interruption of abiraterone acetate, preventing cumulative toxicity of this agent.

## Introduction

Prostate cancer (PC) is, among men, the second common malignancy and the fifth cancer-related leading cause of death worldwide (1). Even if localized PC is treated with a curative intent and excellent outcome, the management of metastatic PC (mPC) remains a challenge for clinicians with a very poor outcome and limited therapeutic options. Metastases from prostate cancer involve mainly the bone compartment and lymph nodes (2). Between the localized and generalized metastatic statuses, oligometastatic disease represents a transition defined by a limited number of metastatic lesions that do not rapidly spread to other sites. Even if this transitional status naturally progresses into disseminated metastatic disease, it could represent a window of opportunity for localized radical treatment. Oligometastatic disease is usually defined by a maximum number of metastatic sites between 3 and 5 (3). However, this definition is largely based on conventional imaging such as bone scan and thoraco-abdominal computed tomography (CT); the increasing use of modern imaging such as PSMA-positron emission tomography (PET) and whole-body magnetic resonance imaging (MRI) will probably allow a better definition of this entity. There are no clear guidelines concerning the management of oligometastatic disease in PC, but many trials are evaluating the role of metastasis-directed therapies (MDT) such as stereotactic body radiotherapy (SBRT) or surgery in this setting (4). Even if MDT could potentially increase progression-free survival (PFS) and delay the initiation of androgen deprivation therapy (ADT) in hormono-sensitive metastatic PC, the role of MDT remains controversial in castration-resistant PC (CRPC) patients with visceral metastases (5, 6). We report the case of a patient with isolated liver metastases progressing on ADT. Could MDT be an option in this patient?

## Case Presentation

**Figure 1:**
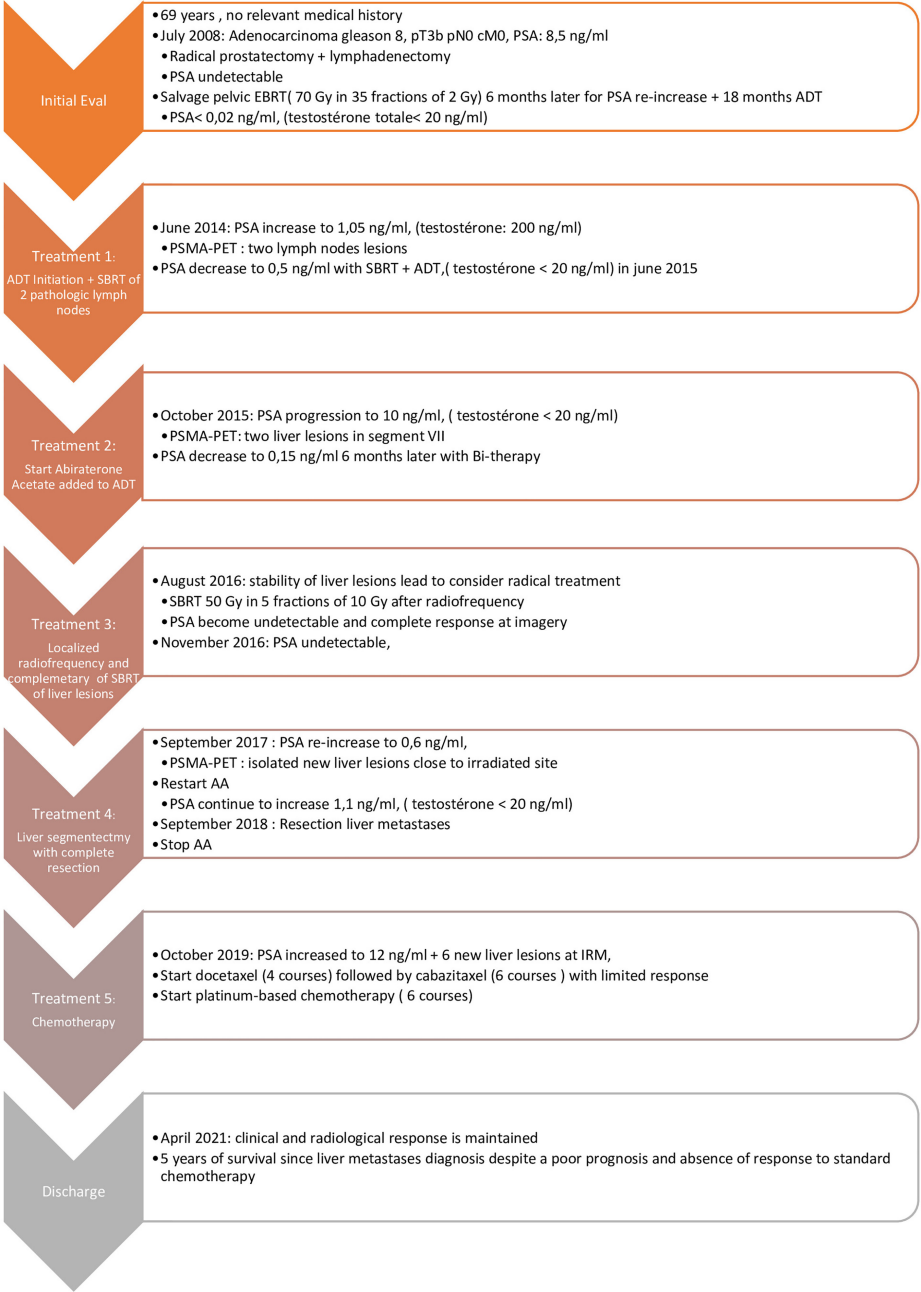
Timeline.

**Figure 2:**
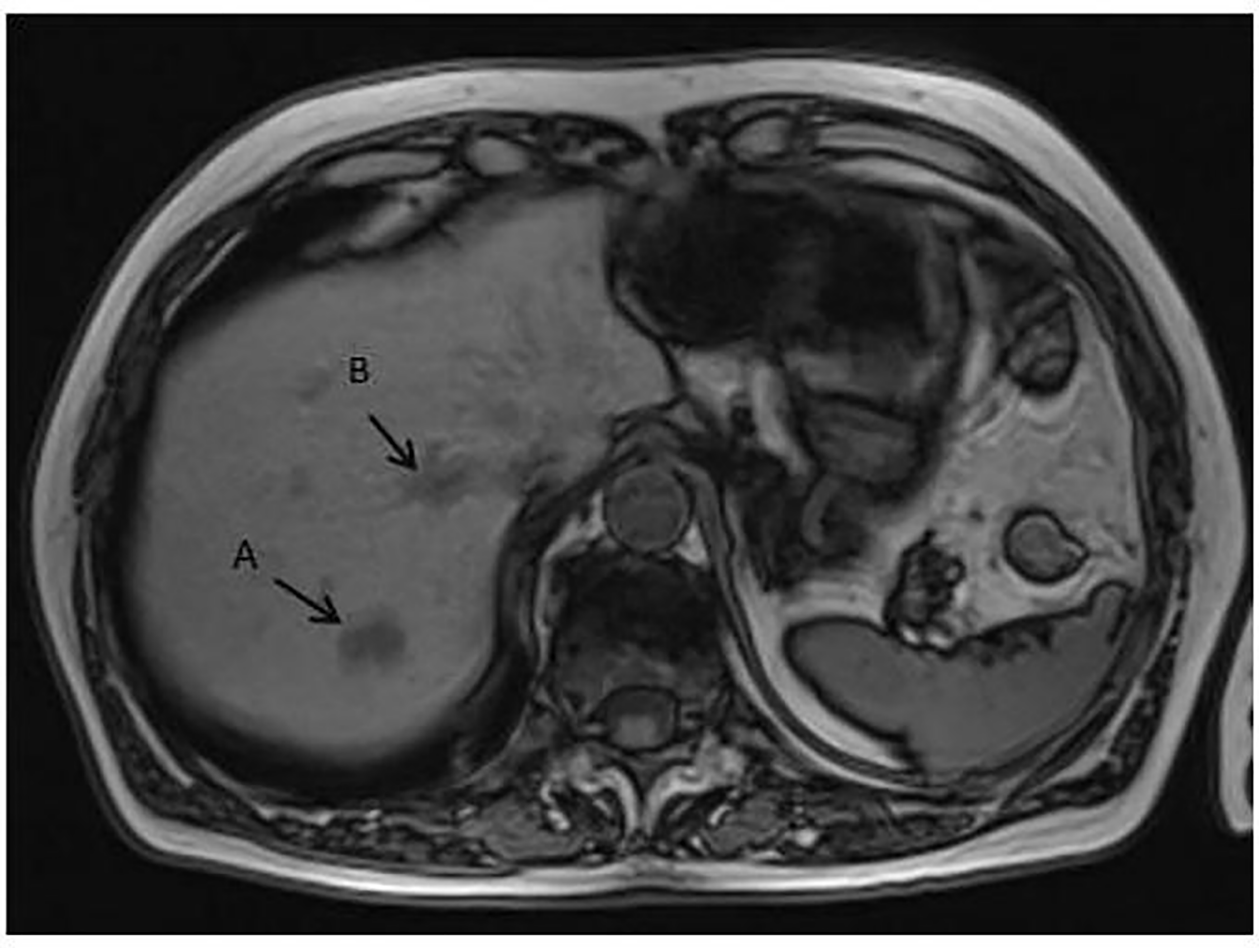
MRI liver revealed two metastases: **(A)** in segment VII and **(B)** straddling segments V and VI.

**Figure 3:**
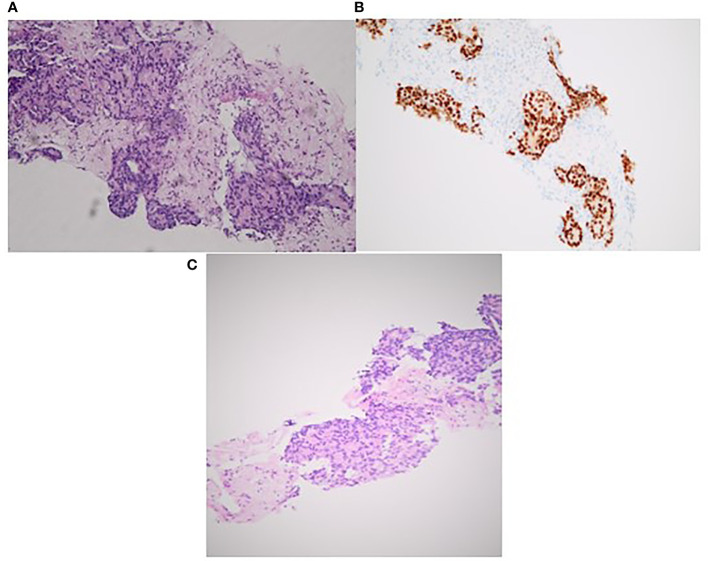
Histopathology findings of liver biopsy lesions confirming prostatic origin with cribriform pattern **(A)**. Positive staining for PSA **(B)**. Hematoxylin and eosin stain (×20) **(C)**.

**Figure 4:**
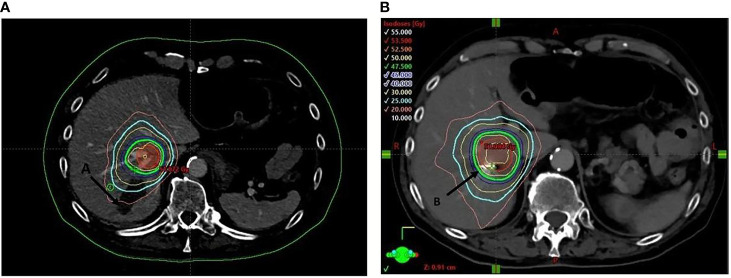
Dose distribution of the stereotactic body of radiotherapy (50 Gy in five fractions) of lesion **(B)** (coils) post radiofrequency of lesion **(A)**.

In July 2008, based on a prostate-specific antigen (PSA) increase (8.5 ng/ml), a 69-year-old man without relevant medical history was diagnosed with a localized prostate adenocarcinoma Gleason 7 (3 + 4). No distant lesion was seen on conventional work-up (bone scan and thoraco-abdominal CT). Radical prostatectomy was performed with lymphadenectomy, confirming Gleason 8 (4 + 4) prostate adenocarcinoma invading the seminal vesicles (cT3bN0M0) (**Figure 1**). Postsurgical PSA was undetectable. Six months later, PSA increased to 0.34 ng/ml and salvage pelvic radiotherapy (70 Gy in 35 fractions of 2 Gy according to the intensity-modulated radiotherapy (IMRT) and an 18-month duration of ADT was performed with a subsequent decrease of PSA (<0.02 ng/ml). In June 2014, PSA re-increased (1.05 ng/ml) with no visible lesion on bone scan and thoraco-abdominal CT; the testosterone level was within normal range (200 ng/dl). A ^68^Gallium (Ga)-PSMA-PET-CT showed two infra-centimetric lymph node lesions (one in the para-rectal area and one in the pre-sacral area). ADT was initiated, and SBRT was performed on these lesions, with a delivered dose of 54 Gy (2 Gy per fraction). PSA decreased progressively with a nadir of 0.5 ng/ml in June 2015 (testosterone <20 ng/dl); ADT was maintained. In October 2015, PSA increased to 10 ng/ml on ADT (testosterone <20 ng/dl) and thoraco-abdominal CT showed one isolated liver lesion. ^68^Ga-PSMA-PET, ^18^Fluorodeoxyglucose (FDG)-PET, and liver magnetic resonance imaging (MRI) showed two liver lesions (lesion A = an 11-mm lesion located in segment VII and lesion B = a 20-mm lesion involving both segments V and VI) (**Figure 2**). Biopsy confirmed prostate adenocarcinoma without any neuroendocrine differentiation (**Figure 3**). Abiraterone acetate plus prednisone (AA-P) was added to ADT and resulted, at 6 months, in a PSA decrease (0.15 and 0.16 ng/ml at 6 and 9 months, respectively) and modest tumor shrinkage (-10% following RECIST criteria at 6 and 9 months, respectively). The limited number of liver lesions, the well-circumscribed aspect of these lesions, the absence of any other visible lesion, and the absence of a more pronounced radiological response led us to consider MDT in addition to ADT and AA-P; in August 2016, microwave needle ablation was performed on lesion A, and 4 weeks later, we performed SBRT (50 Gy in 5 fractions of 10 Gy) on lesion B that was less accessible to radiofrequency (**Figure 4**). This treatment was well tolerated. In November 2016, PSA was undetectable (<0.01 ng/ml) and liver MRI did not show any active lesion, which led us to consider interruption of AA-P and continuation of ADT alone. Nine months after AA-P arrest, PSA re-increased (0.6 ng/ml) and ^68^Ga-PSMA-PET showed a new liver metastasis, close to the irradiated site, without any other distant lesion. AA-P was restarted in October 2017 (testosterone <20 ng/dl) but did not result in a PSA decrease (1.1 ng/ml) or radiological response (5% increase in tumor size, following RECIST criteria) after 9 months; no new lesion was detected on ^68^Ga-PSMA-PET. In front of this maintained radiological stable disease, surgical liver segmentectomy was performed in September 2018; histopathology showed Gleason 8 (4 + 4) prostate cancer adenocarcinoma without neuroendocrine differentiation. Resection was complete, and there were no postoperative complications. AA-P was stopped after surgery while ADT was continued. PSA remained stable at 6 and 9 months (1.5 and 1.9 ng/ml, respectively), and thoraco-abdominal CT did not show any new metastatic lesion at 6 months. However, 12 months after surgery, PSA increased to 12 ng/ml and multiple liver lesions appeared on thoraco-abdominal CT and liver MRI. In September 2019, docetaxel (75 mg/m^2^ every 3 weeks) was started without any radiological response after 3 cycles (progressive disease following RECIST criteria). Six cycles of cabazitaxel (20 mg/m^2^ every 3 weeks) were administered, resulting in a 6-month lasting stable disease. After failure of docetaxel and cabazitaxel, we rapidly initiated platinum-based chemotherapy. After six courses, our patient presented a radiological partial response following RECIST criteria with maintained quality of life. In April 2021, clinical and radiological response was maintained and no treatment had to be reintroduced.

## Discussion and Conclusion

Visceral metastases occur in up to 32% of CRPC patients during disease evolution, involving most commonly the liver and lungs (6, 7). This incidence could increase with time, due to improvement of patient survival and increasing selection of aggressive clones. Liver metastases are associated with poor outcome; in a meta-analysis evaluating 8,820 mCRPC patients treated with docetaxel, the median OS reached 31.6 months in men with lymph node-only disease, 21.3 months in men with non-visceral bone metastases, 19.4 months in men with lung metastases, and 13.5 months in men with liver metastases (2). However, these patients do benefit from conventional treatments including abiraterone, enzalutamide, and chemotherapy (8, 9, 5). MDT of isolated metastases, particularly bone metastases, is emerging as a potential option in PC treatment. Ost et al. showed in a phase II trial (STOMP trial) that MDT could delay CRPC-free survival and the ADT-free survival in hormonosensitive PC with bone or lymph node metastases (6). However, the benefit of MDT remains unclear in the CRPC setting and in liver metastases.

We highlight in this report the feasibility of combining MDT to AA-P in a CRPC patient with isolated liver metastases. The first MDT (SBRT) allowed AA-P interruption during 9 months. At local resurgence, a new MDT strategy (surgery) allowed AA-P arrest and delayed the initiation of a new systemic treatment during 12 months.

We thus showed that, even in liver metastasis and CRPC settings, MDT was feasible and could be considered in order to interrupt systemic treatment and/or decrease cumulative toxicities related to the long use of AA-P. Furthermore, this strategy also delayed the initiation of chemotherapy that, in this case, appeared poorly efficient and that in other patient cases could not be appropriate (low-volume disease in the elderly population, in which docetaxel could deteriorate life quality).

This case highlights the feasibility of combining MDT to AA-P in a CRPC patient with liver metastases and the subsequent possibility to interrupt temporary systemic treatment. Two other options could have been proposed in this patient: the first one was to consider only AA until progression, as recommended by guidelines, and the second one was to continue AA after the first MDT. We do not know whether these options could have led to similar or superior outcomes for our patient. The impact of intermittent AA-P is also not known as no clinical trial has evaluated this strategy. Another limitation is that we mainly based on PSA evolution to decide the MDT strategy or AA-P interruption; further biomarker or imaging tools are needed to correctly define a real oligometastatic status (9–11).

To our knowledge, there are only 10 case reports focusing on the efficacy of MDT in isolated non-lymph-node visceral metastatic lesions (three patients with liver lesions, one patient with cerebral lesions, three patients with testicular lesions, two patients with lung lesions, one patient with testicular and cerebral lesions). MDT consisted in surgical resection for these patients (**Table 1**) (11–20). The radical management of isolated metastases resulted in all these patients in a decrease of PSA; the PFS was equal or superior to 1 year in six patients (and not available in two patients), and OS reached 2 years in four patients (not available in three patients).

**Table 1:** Patient cases reported for metastasis directed therapy in visceral oligometastatic prostate cancer (January 2010–January 2020).

Authors	Initial tumor characteristics	Primary tumor treatment	Progression	Non-visceral metastases	Visceral metastases and clinical features	Metastases management (SBRT/surgery)	Systemic treatment following VM diagnosis	- PSA response- PFS- Systemic treatment- Outcome
Tilmans et al., 2020 (12)	67 years PSA: unknown TNM: unknown Gleason: unknown	- RP - 8 years later: salvage prostatic EBRT + ADT	Metastatic progression on ADT 18 months after onset of EBRT-ADT	None	- 1 liver metastasis - PSA: 32 ng/ml - No neuroendocrine	- Extended left hepatectomy	- ADT - Docetaxel (6 courses every 3 weeks) Before hepatectomy	- PSA < 1 ng/ml - PFS = 1 years - Enzalutamide - OS = 32 months
Ishizaki et al., 2019 (13)	63 years PSA: 9.95 ng/ml T4N1M0 Gleason 5 + 5	- Neo adjuvant ADT + docetaxel (6 courses every 3 weeks) - Prostatic EBRT	Metastatic progression on ADT 22 months after docetaxel	None	- 1 cerebellar metastasis - PSA: 1.34 ng/ml - No neuroendocrine	- Surgical resection + WBRT	- Unknown	- PSA < 1 ng/ml - PFS = 23 months - No systemic treatment - OS = 23 months
Kawai et al., 2017 (14)	55 years PSA: unknown TNM: unknown Gleason: unknown	- RP - Adjuvant ADT - Salvage EBRT 11 years later + ADT (not interrupted since diagnosis)	- Metastatic progression on ADT (never interrupted) 4 years after EBRT	None	- 1 liver metastasis - PSA = 13.77 ng/ml - No neuroendocrine	- Surgical segmentectomy	None	- PSA= 0.54 ng/ml - PFS: 9 months - Docetaxel - OS: NA
Chang et al., 2017 (15)	80 years PSA: unknown TNM: unknown Gleason 4 + 4	- Prostatic EBRT	Metastatic progression 3 years after EBRT	None	-Right testicular metastasis and 1 cerebral metastasis PSA: 319 ng/ml - No neuroendocrine	- Orchiectomy and - 5 fractions of stereotactic brain radiotherapy	None	- PSA decreased to undetectable - PFS = NA - OS = NA
Bonetta et al., 2017 (16)	58 years PSA: 7.6 ng/ml pT3bN0M0 Gleason 4 + 5	- RP - Adjuvant RT - ADT declined by the patient	Metastatic Progression 32 months after RT	None	- Left testicular metastasis - PSA: 0.61 ng/ml - No neuroendocrine	- Orchidectomy	None (ADT declined by the patient)	- PSA decreased to 0.01 ng/ml. - PFS ≥ 5 years - No new systemic treatment - OS ≥ 5 years
Wang et al., 2016 (17)	68 years PSA: 7.6 ng/ml pT3aN0M0 Gleason 3 + 4	-EBRT+ 18-month ADT	Metastatic Progression 6 years after end of ADT	None	- 1 liver metastasis - PSA: 48 ng/ml - No neuroendocrine	- Left hepatic lobectomy	ADT	- PSA decreased to < 0.01 ng/ml - PFS ≥ 1 year - No new systemic treatment - OS ≥ 1 year
Peres Gago et al., 2016 (18)	55 years PSA: 4.5 ng/ml pT3aNX Gleason 4 + 3	- RP - Salvage EBRT 2 years later (No ADT)	Progression 22 months after EBRT	None	- Lung nodules - PSA: NA - No neuroendocrine	- Surgical resection	ADT	- PSA decreased to < 0.01 ng/dl - PFS ≥ 4 years - No new systemic treatment - OS ≥ 4 years
Wallis CJD et al., 2011 (19)	46 years PSA: 14.7 ng/ml pT3aN0M0 Gleason 4 + 5	- Neo-adjuvant ADT - RP - Salvage EBRT 6 years later (no ADT)	Metastatic progression 6 months after EBRT	None	- 3 lung nodes - PSA: NA - No neuroendocrine	- Surgical resection	Unknown	- PSA decreased to 0.28 ng/ml - PFS: 9 months - No new systemic treatment - OS > 1 years
Kwon et al., 2011 (20)	66 years PSA: unknown pT3N0M0 Gleason 4 + 5	- RP - Adjuvant EBRT + ADT	Metastatic progression on ADT 4 months after EBRT	None	- Testicular metastasis - PSA: 0.347 ng/ml - No neuroendocrine feature	- Orchidectomy	ADT continuation	- PSA decreased to 0.03 ng/ml - PF = NA - OS = NA
Janssen et al., 2010 (21)	68 years PSA: 7.66 ng/ml pT3bpN0M0 Gleason 3 + 3 Cribriform feature	- Neo adjuvant ADT - RP - Salvage EBRT 2.5 years later	Metastatic Progression 1 month after end of RT	None	- Testicular metastasis - PSA: 3.08 ng/ml - No neuroendocrine feature	- Orchiectomy	None	- PSA decreased to 0.07 ng/dl - PFS ≥ 2 years - No new systemic treatment - OS ≥ 2 years

EBRT, external beam radiotherapy; RP, radical prostatectomy; HT, hormone therapy; ADT, androgen deprivation therapy; PND, pelvic node dissection; LFT, liver function test; Pca, prostate cancer; PFS, progression-free survival; WBRT, whole-brain radiotherapy; OS, overall survival.

This case report suggests the feasibility to combine MDT to systemic treatment in poor prognosis mCRPC patients. Increasing evidence shows the efficacy of MDT in delay systemic treatment onset in the early mPC stage (metastatic hormonosensitive PC). We suggest that this strategy could also be considered very early in CRPC patients particularly if MDT could allow systemic treatment interruption, prevent cumulative toxicities, and delay subsequent line of treatment.

## Data Availability Statement

The original contributions presented in the study are included in the article/supplementary material. Further inquiries can be directed to the corresponding author.

## Ethics Statement

Written informed consent was obtained from the individual(s)’ and minor(s)’ legal guardian/next of kin, for the publication of any potentially identifiable images or data included in this article.

## Author Contributions

A-EY analyzed all the data and was a major contributor in writing the manuscript. AH performed the oncologic treatment and contributed to the writing manuscript. CC performed the oncologic treatment and contributed to the writing of the manuscript. NC performed the SBRT treatment and contributed to the writing of the manuscript. BM performed the liver surgery and contributed to the writing of the manuscript. GP performed the histopathology analysis and contributed to the writing of the manuscript. ES performed the oncologic treatment and was a major contributor in writing the manuscript. All authors contributed to the article and approved the submitted version.

## Conflict of Interest

The authors declare that the research was conducted in the absence of any commercial or financial relationships that could be construed as a potential conflict of interest.

## Publisher’s Note

All claims expressed in this article are solely those of the authors and do not necessarily represent those of their affiliated organizations, or those of the publisher, the editors and the reviewers. Any product that may be evaluated in this article, or claim that may be made by its manufacturer, is not guaranteed or endorsed by the publisher.

## Abbreviations

AA-P, abiraterone acetate prednisone; ADT, androgen deprivation therapy; mCRPC, metastatic castration-resistant prostate cancer; MDT, metastasis-directed therapies; mPC, metastatic prostate cancer; MRI, magnetic resonance imaging; PC, prostate cancer; PET, positron emission tomography; PSA, prostate-specific antigen; SBRT, stereotactic body radiotherapy.
